# Enhanced differentiation of the mouse oli-neu oligodendroglial cell line using optimized culture conditions

**DOI:** 10.1186/s13104-023-06432-w

**Published:** 2023-08-04

**Authors:** Guillermo Rodriguez Bey, Quasar Saleem Padiath

**Affiliations:** 1 Department of Human Genetics, School of Public Health, University of Pittsburgh, 130 De Soto St, Pittsburgh, PA 15261 USA; 2 Department of Neurobiology, School of Medicine, University of Pittsburgh, Pittsburgh, PA USA

**Keywords:** Oligodendrocyte, Cell culture, Myelin, Oli-neu

## Abstract

**Objective:**

Oligodendrocytes (OL) are the glial cell type in the CNS that are responsible for myelin formation. The ability to culture OLs in vitro has provided critical insights into the mechanisms underlying their function. However, primary OL cultures are tedious to obtain, difficult to propagate and are not easily conducive to genetic manipulation. To overcome these obstacles, researchers have generated immortalized OL like cell lines derived from various species. One such cell line is the mouse Oli-neu line which is thought to recapitulate characteristics of OLs in early stages of maturity. They have been extensively utilized in multiple studies as surrogates for OLs, especially in analyzing epigenetic modifications and regulatory pathways in the OL lineage.

**Results:**

In this report we present the development of optimized culture media and growth conditions that greatly facilitate the differentiation of Oli-neu cells. Oli-neu cells differentiated using these new protocols exhibit a higher expression of myelin related genes and increased branching, both of which are defining characteristics of mature OLs, when compared to previous culture protocols. We envision that these new culture conditions will greatly facilitate the use of Oli-neu cells and enhance their ability to recapitulate the salient features of primary OLs.

**Supplementary Information:**

The online version contains supplementary material available at 10.1186/s13104-023-06432-w.

## Introduction

Myelin is a primarily vertebrate adaptation that allows for an increase in the rate of axonal condition without necessitating a concomitant increase in axonal diameter [1, 2]. In the central nervous system (CNS), myelin is a dramatic modification of the oligodendrocyte (OL) cell membrane that wraps around an axonal segment in a concentric spiral manner [2, 3].

OLs are derived from oligodendrocyte precursor cells (OPCs) which, in turn, arise from neuroepithelial cells [4, 5]. The differentiation of OPCs into mature myelinating OLs is tightly regulated and involves a complex process whereby OPCs are transformed from bipolar, highly proliferative, migratory cells expressing specific cell surface markers such as Platelet derived growth factor alpha (PDGFRα) into terminally differentiated non-proliferating and highly branched OLs that express myelin specific proteins such as Myelin Basic Protein (MBP), Proteolipid Protein type 1 (PLP1) and 2’,3’-cyclic nucleotide 3’ phosphodiesterase (CNP) [6–8].

In vitro studies of OPCs and OLs have been critical to further understanding the physiology of these cell types and identifying cellular and genetic mechanisms underlying their function. In the case of mice, OL lineage cells are usually isolated from whole brain preparations of early post-natal animals either as OPCs, or directly as mature OLs [9]. In both cases, methods to extract these cell types can be tedious and time consuming and necessitate the ability to work with live mice. In addition, once differentiated into mature OLs, these cells might only survive for short periods of time, usually not exceeding one week [10]. Genetic manipulation such are transfection for the introduction of exogenous gene expression or RNAi treatment and CRISPR based genome editing can also be difficult and of a very low efficiency unless specialized viral vectors are utilized [11, 12].

An alternative approach for in vitro studies has been to utilize immortalized cell lines derived from OL lineage cells. Although these cell lines do not recapitulate all features of OPCs and mature OLs, they have been widely used because of their ease of growth and robust tolerance of various genetic manipulations [13]. One such cell type is the mouse Oli-neu immortalized cell line which was generated by the introduction of the *t-neu* oncogene into a mixed primary culture of oligodendrocytes and their precursor cells [14]. These cells possess many characteristics of OL lineage cells and undifferentiated Oli-neu cells are considered similar to OPCs while differentiated Oli-neu cells are thought to recapitulate OLs in early stages of maturity [13]. They have been extensively utilized in multiple studies as surrogates for early stage OLs, especially in analyzing regulatory pathways in the OL lineage and to model OL and myelin related disease phenotypes [15, 16].

In this report, we describe an optimized culture medium and culture conditions that greatly enhance the differentiation of Oli-neu cells as evidenced by significantly increased myelin gene expression and cellular arborization. This protocol has been utilized in our recent publication where we used Oli-neu cells to model human mutations associated with a demyelination phenotype [17]. However, a detained comparison and description of the enhanced efficiency of the current approach was not carried out and is presented below.

## Methods

### Cell culture

Undifferentiated Oli-neu cells were proliferated at 37 °C in 5% CO2 in culture medium previously described for the culture of OPCs [9]. This medium consisted of SATO media supplemented with platelet derived growth factor (PDGF, 20 ng/ml, PeproTech), Neurotrophin-3 (NT-3, 1 ng/ml, PeproTech), Ciliary Neurotrophic Factor (CNTF, 10 ng/ml, PeproTech) and Forskolin (4.2 ng/ml). Cells were subsequently switched to two types of differentiation medium for 3 days. The previously described ‘standard’ Oli-neu differentiation medium consisted of SATO medium supplemented with 1% horse serum, gentamycin and 1 mM dibutyryl cAMP (dbcAMP, PeproTech) [18]. Our ‘optimized’ differentiation SATO medium was similar to the proliferation medium with the addition of 0.1X B-27 (50X stock, PeproTech), 3,3′,5-Triiodo-L-thyronine (T3, 8 ng/ml, PeproTech) and PD 174,265 (1 nM, Cayman chemical) and omission of PDGF. This culture medium is similar to that used to differentiate primary OLs [9] with the major differences being a reduction in the B27 concentrations from 1X to 0.1X and a doubling of the T3 concentration in our medium.

### Quantitative real-time reverse transcriptase PCR (qRT-PCR)

Total RNA was extracted from Oli-neu cells using TRIzol (Ambion). After treatment with DNAase (Invitrogen), one µg of RNA was used for cDNA synthesis with qScript™ cDNA SuperMix (Quanta). RNA was extracted from 3 independent biological replicates for each condition, QT-PCR was performed on an ABI 7900HT real-time thermocycler (Life Technologies) and analyzed using the ∆∆CT method [19] and normalized to *Gapdh* as the internal control. PCR primers utilized are described in Supplementary Tables.

### Immunoblotting

Total protein was extracted from Oli-neu cells using T-PER buffer (Thermo Scientific) with a protease inhibitor cocktail (Roche). Protein was extracted from 3 independent biological replicates for each condition. Proteins were separated by acrylamide gel electrophoresis and transferred to nitrocellulose membranes followed by blocking with Odyssey Blocking Buffer (Licor) in TBS for 1 h at room temperature. Membranes were blotted with specific antibodies against CNP and GAPDH after washing in 1x TBS-T overnight at 4 °C. Primary antibodies were diluted in 0.2% Tween 20/Licor TBS buffer. Secondary antibodies were incubated for 1 h at room temperature. Blots were imaged using an Odyssey CLx Imaging System (Licor). Antibodies used have been described in supplementary material. The uncropped immunoblot is shown in the supplementary figure.

### Immunocytochemistry

Oli-neu cells were analyzed by Immunocytochemistry (ICC) using antibodies against β Tubulin and CNP, following standard protocols. Cells were grown in Poly-D-lysine treated coverslips and fixed in ice cold absolute methanol for 25 min. Phosphate Buffered Saline (PBS) buffer washes were followed by blocking with 1% in normal donkey serum (Jackson Immunoresearch) and 0.3% of Triton X-100 for one hour at room temperature. We incubated the primary antibodies in 1% Bovine Serum Albumin (BSA) and 0.3% Triton X-100 in PBS overnight at 4 °C. After washing in PBS, fluorescent conjugated secondary antibodies were incubated for one hour at room temperature. Slides were mounted were using antifade mounting medium with DAPI (Vectashield). Imaging was carried out a Leica CTR5000 epifluorescence microscope. For analysis, different fields were used for each coverslip. Three independent coverslips, derived from three independent cell seedings/platings (independent biological replicates) were analyzed. The investigator was blinded during the analysis.

All antibodies for Immunoblotting and ICC have been listed in Supplementary Tables.

## Results

**Fig. 1:**
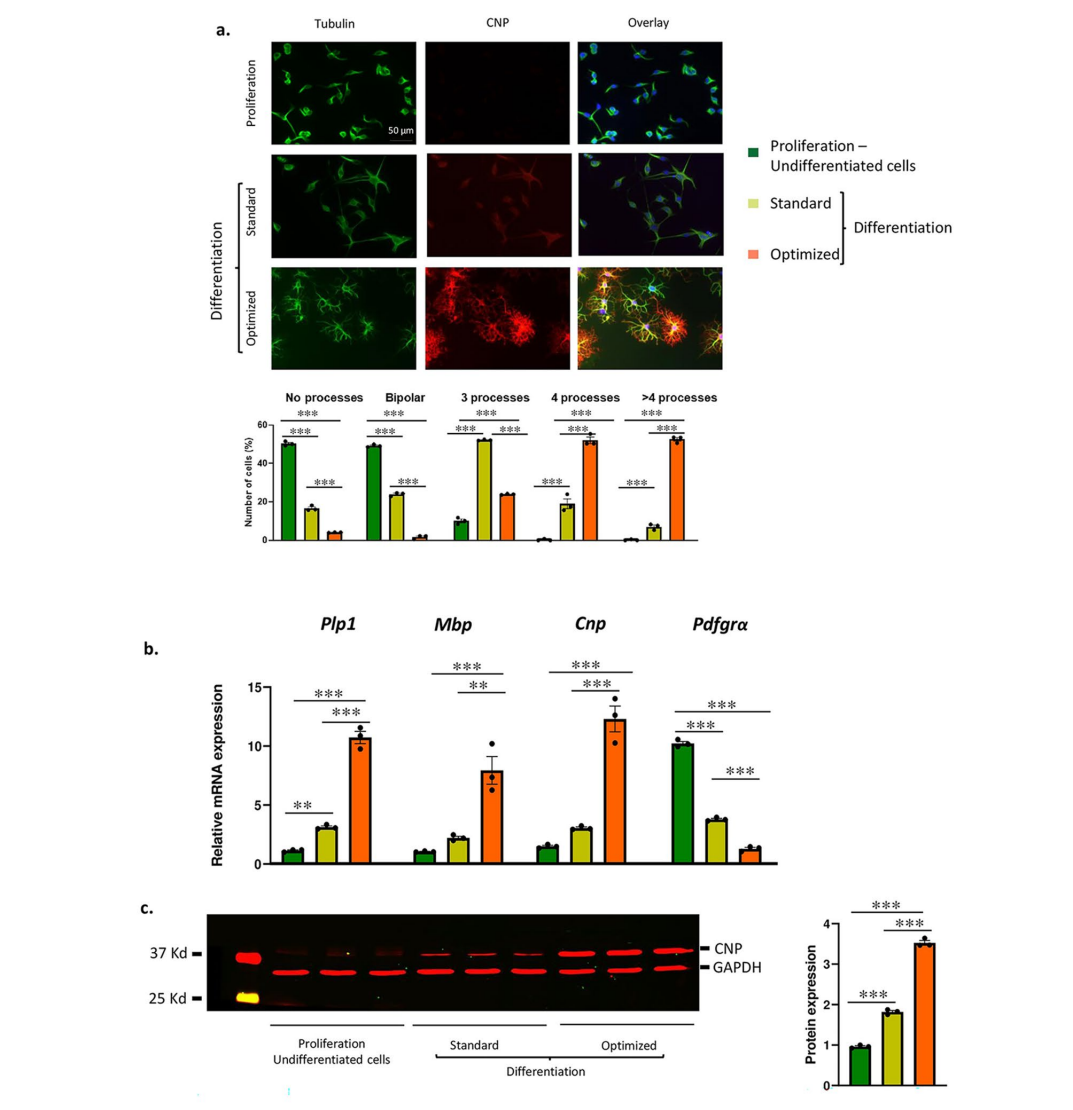
Oli-neu cultured in optimized media exhibits enhanced differentiation: **(A)** ICC analysis of Oli-neu cells in the various culture media stained with antibodies against β tubulin (green channel) and CNP (red channel). β tubulin allows the identification of cytoplasmic arborization while CNP localizes to the cytoplasm but is only expressed in mature OL lineage cells. Top panels – undifferentiated Oli-neu cells in proliferation medium. Middle and lower panels – Oli-neu cells cultured in standard and optimized differentiation medium, respectively. Quantification of proportion of the cells with more than two processes. Significant increases in arborization were observed in cells cultured in optimized differentiation medium. Error bars are 95% confidence intervals from more than 200 cells counted for each biological replicate, ***P < 0.001, calculated by ANOVA with post-hoc Tukey test (Scale bar − 50 µM).**(B)** mRNA expression of myelin specific genes, *Plp1, Mbp and Cnp*, and a marker for OPCs, *Pdgfrα*, in Oli-neu cells grown in different media. Expression of individual genes was analyzed by real time PCR and normalized to *Gapdh* as an internal control and plotted as a ratio of expression in undifferentiated cells. Expression was significantly increased for all three myelin specific genes but decreased for *Pdgfrα* in cells grown in the optimized differentiation medium. Error bars are standard error of the mean, n = 3 independent biological replicates, *p < 0.05 **p < 0.01, ***p < 0.001 analyzed by ANOVA with post-hoc Tukey test. **(C)**. Representative immunoblot of protein lysates from Oli-neu cells cultures in the three different media. Immunoblots were simultaneously probed with anti-CNP (red) and anti-GAPDH (red) as a loading control. Quantitation of CNP relative to GAPDH show significant increases in CNP expression in cells grown in optimized medium (n = 3 independent biological replicates, **p < 0.01, ***p < 0.001 analyzed by ANOVA with post-hoc Tukey test

Oli-neu cells can be maintained in an undifferentiated state where they proliferate and exhibit properties similar to OPCs. Switching undifferentiated Oli-neu cells from proliferation to differentiation medium prevents further cell division promotes the maturation into OL type cells [20]. Once switched to the differentiation media, Oli-neu cells recapitulated the morphological transitions from OPCs to OLs and exhibited dramatic increases in the number of cytoplasmic processes and arborization, especially in our optimized differentiation medium. Differentiated Oli-neu cells cultured using our optimized protocol revealed significant increases in the fraction of cells with multiple cytoplasmic processes when compared to the standard media. In cells cultured in the optimized medium, 87.5% exhibited ≥ 4 processes, while this was only 19.8% in the standard medium and 1% in the undifferentiated cells. These results indicated that a higher percentage of cells had undergone differentiation using the optimized protocol (Fig. 1a). Consistent with this observation, we detected significantly higher mRNA expression levels of the myelin genes *Mbp*, *Plp1* and *Cnp* when compared to the cells differentiated using the standard media and undifferentiated cells (Fig. 1b). We also observed a greater reduction in *Pdgfrα *mRNA, a marker for OPCs [8], using the optimized differentiation medium (Fig. 1b). Previous reports have demonstrated that differentiated Oli-neu cells express these myelin genes only at the RNA level with the exception of CNP, that is also expressed at the protein level [13]. Immuno-blot analysis revealed significant increases in CNP protein expression when compared to standard media and undifferentiated cells (Fig. 1c).

All these results taken together indicate that culturing Oli-neu cells in our optimized medium greatly enhances differentiation of Oli-neu cells into mature OL type cells. A possible mechanism for the improved efficiency of our optimized medium is that the reduction of B27 and increase in T3 might accelerate the exit of proliferating Oli-neu cells from the cell cycle thereby facilitating differentiation. We anticipate that the use of this medium will facilitate a more efficient differentiation of Oli-neu cells and greatly enhance their utility in the study of OL regulation and function.

### Limitations

While the current protocol clearly improves the differentiation efficiency of Oli-neu cells as evidenced by myelin gene expression and increased branching, a more detailed analysis using genome wide approaches such as RNA Seq would be required to compare the full extent of the similarity between primary OLs and the Oli-neu cells derived using the protocol we have described. Although Oli-neu cells appear to be more differentiated, they are still not perfect surrogates for primary mature OLs as they do not express many of the myelin gens at the protein level and cannot myelinate axons *in vitro.*

### Electronic supplementary material

Below is the link to the electronic supplementary material.

Supplementary Material 1

Supplementary Material 2

## Data Availability

All data that support the findings of this study are available from the corresponding author upon reasonable request.

## References

[1] Snaidero N, Simons M (2014). Myelination at a glance. J Cell Sci.

[2] Verkhratsky A, Butt A. Glial physiology and pathophysiology. John Wiley & Sons, Ltd; 2013. pp. 245–320.

[3] Snaidero N, Mobius W, Czopka T, Hekking LH, Mathisen C, Verkleij D (2014). Myelin membrane wrapping of CNS axons by PI(3,4,5)P3-dependent polarized growth at the inner tongue. Cell.

[4] Goldman SA, Kuypers NJ (2015). How to make an oligodendrocyte. Development.

[5] Rowitch DH, Kriegstein AR (2010). Developmental genetics of vertebrate glial-cell specification. Nature.

[6] Emery B, Lu QR (2015). Transcriptional and epigenetic regulation of Oligodendrocyte Development and Myelination in the Central Nervous System. Cold Spring Harb Perspect Biol.

[7] Barateiro A, Brites D, Fernandes A (2016). Oligodendrocyte Development and Myelination in Neurodevelopment: Molecular Mechanisms in Health and Disease. Curr Pharm Des.

[8] Zhang SC (2001). Defining glial cells during CNS development. Nat Rev Neurosci.

[9] Emery B, Dugas JC (2013). Purification of oligodendrocyte lineage cells from mouse cortices by immunopanning. Cold Spring Harb Protoc.

[10] Hyrien O, Mayer-Proschel M, Noble M, Yakovlev A (2005). Estimating the life-span of oligodendrocytes from clonal data on their development in cell culture. Math Biosci.

[11] Krueger WH, Madison DL, Pfeiffer SE (1998). Transient transfection of oligodendrocyte progenitors by electroporation. Neurochem Res.

[12] Gresch O, Altrogge L (2012). Transfection of difficult-to-transfect primary mammalian cells. Methods Mol Biol.

[13] Pereira GB, Dobretsova A, Hamdan H, Wight PA (2011). Expression of myelin genes: comparative analysis of Oli-neu and N20.1 oligodendroglial cell lines. J Neurosci Res.

[14] Jung M, Kramer E, Grzenkowski M, Tang K, Blakemore W, Aguzzi A (1995). Lines of murine oligodendroglial precursor cells immortalized by an activated neu tyrosine kinase show distinct degrees of interaction with axons in vitro and in vivo. Eur J Neurosci.

[15] Kim D, An H, Shearer RS, Sharif M, Fan C, Choi JO (2019). A principled strategy for mapping enhancers to genes. Sci Rep.

[16] Curiel J, Rodriguez Bey G, Takanohashi A, Bugiani M, Fu X, Wolf NI (2017). TUBB4A mutations result in specific neuronal and oligodendrocytic defects that closely match clinically distinct phenotypes. Hum Mol Genet.

[17] do Rosario MC, Bey GR, Nmezi B, Liu F, Oranburg T, Cohen ASA et al. Variants in the zinc transporter TMEM163 cause a hypomyelinating leukodystrophy. Brain. 2022. 10.1093/brain/awac295 PMC10200305 35953447

[18] Sohl G, Hombach S, Degen J, Odermatt B (2013). The oligodendroglial precursor cell line Oli-neu represents a cell culture system to examine functional expression of the mouse gap junction gene connexin29 (Cx29). Front Pharmacol.

[19] Livak KJ, Schmittgen TD (2001). Analysis of relative gene expression data using real-time quantitative PCR and the 2(-Delta Delta C(T)) method. Methods.

[20] Myster DL, Duronio RJ (2000). To differentiate or not to differentiate?. Curr Biol.

