# Supplementary material for: Enhanced differentiation of the mouse oli-neu oligodendroglial cell line using optimized culture conditions
Source: BMC Res Notes. 2023 Aug 4;16:161. doi: 10.1186/s13104-023-06432-w (PMC10401818; doi:10.1186/s13104-023-06432-w)
Supplement: Supplementary file 1 — Supplementary Material 1 [file 13104_2023_6432_MOESM1_ESM.docx]

**Supplementary information**

Real time PCR primers utilized in the study

| **Gene** | **Forward** | **Reverse** |  | | |
| --- | --- | --- | --- | --- | --- |
|  |  |  | |  |  |
| *Plp1* | GCCCCTACCAGACATCTAGC | AGTCAGCCGCAAAACAGACT | |  | |
| *Mbp* | CTCACACACGAGAACTAC | CTTGAAGAAATGGACTACTG | |  | |
| *Cnp* | GTTCTGAGACCCTCCGAAAA | CCTTGGGTTCATCTCCAGAA | |  | |
| *Pdgfrα* | GGAAGGACTGGAAGCTTGGGGC | GAGATGAGGCCCGGCCCTGTGA | |  | |
| *Gapdh* | AACAGCAACTCCCACTCTTC | CCTGTTGCTGTAGCCGTATT | |  | |
|  |  |  | |  | |

Antibodies utilized in the study

Primary antibodies

|  |  |  |  | **Dilution** | |
| --- | --- | --- | --- | --- | --- |
|  | **Company** | **Ref.** | **Species** | **WB** | **IHC** |
| TUB | Millipore | MAB1864 | Rat | - | 1/250 |
| CNP | Millipore | NE1020 | Mouse | 1/500 | 1/250 |
| GAPDH | ThermoFisher | MA5-15738 | Mouse | 1/5000 | - |

Secondary antibodies:

For WB, we used as secondary antibody, IRDye 680LT (Licor) in the red channel for mouse antibodies (CNP and GAPDH.

For IHC, We used Cy3 (Jackson IR, 715-165-150) for CNP and AF488 (Jackson IR, 712-545-150) for Tubulin.
