# Supplementary figures and images for: Enhanced differentiation of the mouse oli-neu oligodendroglial cell line using optimized culture conditions
Source: BMC Res Notes. 2023 Aug 4;16:161. doi: 10.1186/s13104-023-06432-w (PMC10401818; doi:10.1186/s13104-023-06432-w)

75 kDa

25 kDa

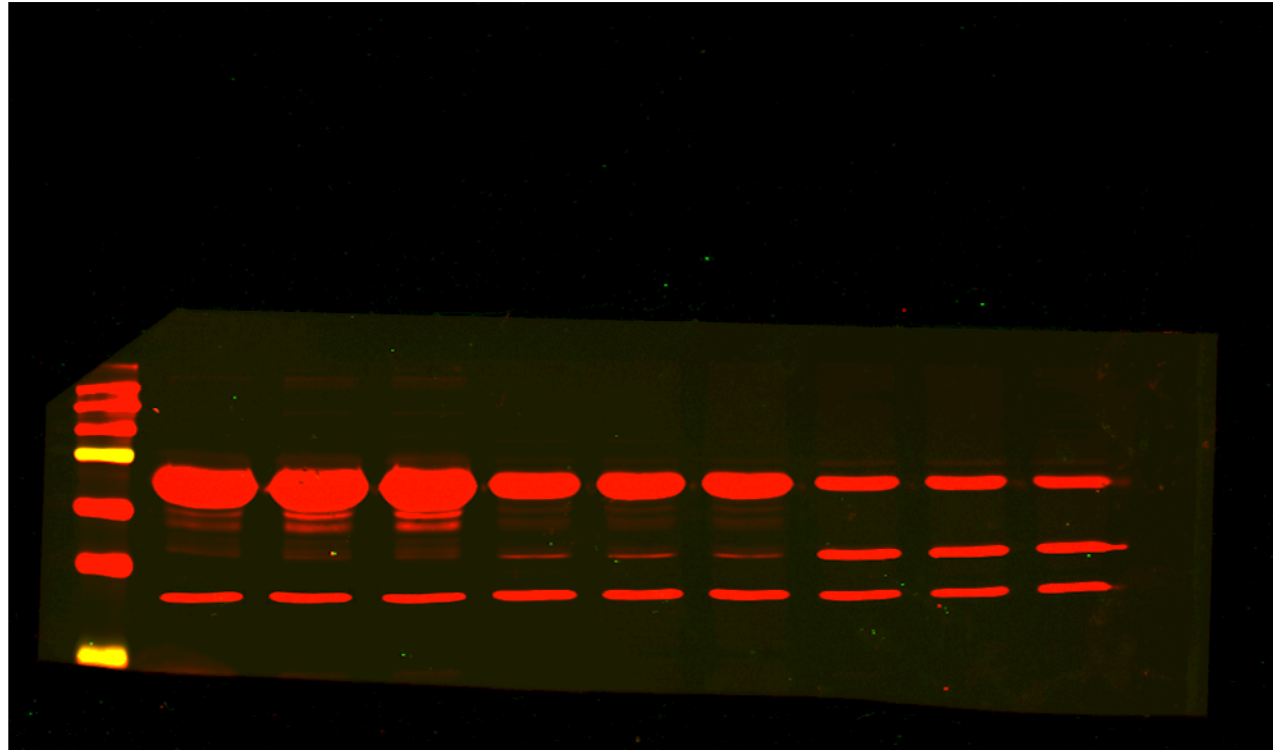

— Non Specific band

— CNP

— GAPDH

Supplement: Supplementary file 2 — Supplementary Material 2 [file 13104_2023_6432_MOESM2_ESM.pdf]
